# A WAV file dataset of bottlenose dolphin whistles, clicks, and pulse sounds during trawling interactions

**DOI:** 10.1038/s41597-023-02547-8

**Published:** 2023-09-22

**Authors:** Francesco Di Nardo, Rocco De Marco, Alessandro Lucchetti, David Scaradozzi

**Affiliations:** 1 Dipartimento di ingegneria dell’informazione, Università Politecnica delle Marche, Ancona, Italy; 2 Institute of Biological Resources and Marine Biotechnology (IRBIM), National Research Council (CNR), Ancona, Italy; 3 National Biodiversity Future Center, Palermo, Italy

**Keywords:** Marine biology, Animal behaviour, Scientific data

## Abstract

Globally, interactions between fishing activities and dolphins are cause for concern due to their negative effects on both mammals and fishermen. The recording of acoustic emissions could aid in detecting the presence of dolphins in close proximity to fishing gear, elucidating their behavior, and guiding potential management measures designed to limit this harmful phenomenon. This data descriptor presents a dataset of acoustic recordings (WAV files) collected during interactions between common bottlenose dolphins (*Tursiops truncatus*) and fishing activities in the Adriatic Sea. This dataset is distinguished by the high complexity of its repertoire, which includes various different typologies of dolphin emission. Specifically, a group of free-ranging dolphins was found to emit frequency-modulated whistles, echolocation clicks, and burst pulse signals, including feeding buzzes. An analysis of signal quality based on the signal-to-noise ratio was conducted to validate the dataset. The signal digital files and corresponding features make this dataset suitable for studying dolphin behavior in order to gain a deeper understanding of their communication and interaction with fishing gear (trawl).

## Abstract (summary)

**Table:** 

**Measurement(s)**	Cetaceans’ depredation
**Measurement Type(s)**	Vocalization behavior
**Technology Type(s)**	Monitoring – Data acquisition
**Sample Characteristic - Organism**	Dolphins - *Tursiops truncatus*
**Sample Characteristic - Environment**	Sea
**Sample Characteristic - Location**	North-central Adriatic Sea

## Background & Summary

In recent years, the international scientific community has intensified efforts to better understand the interactions between fishing activities and marine mammals in order to identify potential technical or management solutions^1,2^. The feeding behavior of dolphins causes them to interact intensively with small coastal fisheries operating with passive nets (gillnets and trammel nets) and larger commercial vessels operating with bottom and pelagic trawls^1–3^. This interaction with fishing gear by cetaceans, particularly dolphins, may have implications for the conservation of these marine mammals as well as negative economic and social repercussions for fishing operators. Specifically, cetaceans could (*i*) become entangled in nets while attempting to prey on fish that have already been caught (bycatch); (*ii*) injure themselves by bumping into the nets or other components of the fishing gear, which could have fatal consequences for the specimens concerned; and (*iii*) ingest pieces of the net during depredation, which could result in fatal oesophageal or intestinal occlusions. In turn, cetaceans may impact fisheries by (*i*) removing fish already caught from the nets; (*ii*) breaking nets in the attempt to catch fish^3,4^, resulting in further economic losses due to the need to replace or repair the gear^5^; (*iii*) ruining the caught fish by beheading or skinning them, thereby rendering them unmarketable; and (*iv*) scattering the fish in an area, thereby reducing the fishery’s efficiency^5^. In the context of the Mediterranean, the common bottlenose dolphin (*Tursiops truncatus*) is identified as the primary concern for the fishing industry^2^. This species is accustomed to interacting with different types of gear used in commercial, artisanal, and recreational fishing and is capable of developing advanced learning skills for fishing activities (i.e., selecting which vessels to follow, feeding on catch lost during hauling, and/or scavenging discarded catch)^2^.

Assessing depredation events and identifying measures to sustainably mitigate the associated damage caused or endured by dolphins requires an in-depth understanding of dolphins’ habits, movements, interactions with nets, and communication between cetaceans (social life). Therefore, one of the primary goals of marine monitoring is the collection, storage, analysis, and interpretation of underwater bioacoustic signals^6–8^. Specifically, the identification of spatial and temporal patterns in these acoustic signals could help characterize certain dolphin living conditions^9–11^. This applies in general, but especially to the description of the depredation event, in order to document the presence of dolphins in the vicinity of nets and increase knowledge of dolphin predation behavior near fishing gear. Understanding the elements that drive interactions between dolphins and fishing activities is essential for the development of effective mitigation strategies.

The acoustic repertoire of common bottlenose dolphins is characterized by a high degree of complexity^10,11^, including vocal learning, i.e., the ability to tune and produce new vocalizations through experience, and vocal mimicry, i.e., the ability to imitate sound patterns^12^. This species typically emits three main types of acoustic signals: frequency-modulated whistles, echolocation clicks, and multiple burst pulse signals. Narrow-band frequency-modulated whistles are typically interpreted as communication signals, and dolphin socialization increases emissions^13^. This type of emission is associated with activities such as individual identification, coordination of group activities, and the organization of group movements. Typically, whistle characteristics are quantified using standard parameters such as temporal duration and frequency content^14^. Dolphins emit highly directional echolocation click trains during navigation and hunting in order to detect, identify, and differentiate objects and individuals of interest^15^. Due to their high frequency of occurrence during echolocation, these signals are typically analyzed to assess the daily and seasonal presence of dolphins in a given area^16^. Burst pulse sounds, on the other hand, consist of broadband pulses (mainly ultrasonic) with similar characteristics to echolocation clicks. However, they differ from echolocation clicks in their frequency (which is lower) and the duration of the intervals between clicks (which is shorter). Attempts to classify burst pulse sounds based on behavioral context analysis are reported in the literature^17–19^. Nonetheless, researchers disagree on the interpretation; therefore, this is still a matter of debate.

Although bottlenose dolphin emissions are extensively documented^20^, there is a dearth of literature that focuses on the collection, analysis, and interpretation of signals during the interaction between these cetaceans and fishing activities, notably during depredation. However, from an acoustic point of view, the analysis of the depredation event could be particularly relevant, as it is characterized by long-duration interactions between dolphins and fishing activities (and thus protracted activity), as well as a high density of animals and sound emissions. Therefore, the aim of this study is to present a dataset of raw broadband sound recordings of common bottlenose dolphin vocalizations during interactions with fishing activities in the Adriatic Sea, particularly during depredation. In addition to the availability of the complete raw audio signal, a conventional analysis of dolphin sounds^21^ was conducted by visual inspection to identify the frequency-modulated whistles supplied in this paper. Moreover, a literature-based analysis^18^ of the acoustic signal was carried out in order to provide information on the quantitative evaluation of the echolocation clicks and pulse sounds detected in the recordings.

## Methods

### Data collection

Through the active collaboration of researchers and fishermen, data were collected during fishing operations that formed part of the experimental campaign conducted within the framework of the EU Life Delfi project [Project–Dolphin Experience: Lowering Fishing Interactions (LIFE18NAT/IT/000942), www.lifedelfi.eu], which aims to introduce new technical solutions to control and reduce the interaction between marine mammals and professional fishing activities.

**Fig. 1:**
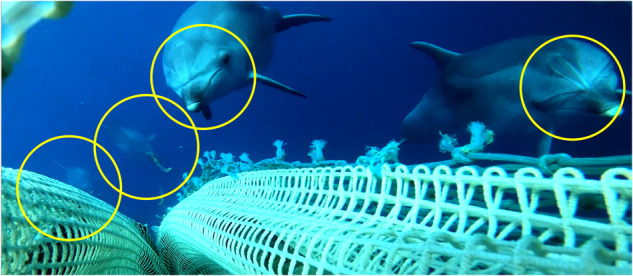
Image captured by a video camera positioned on the trawl net during the recording session. Yellow circles highlight the presence of at least four dolphins.

The signals were recorded in autumn 2021 in the north-central Adriatic Sea during fishing operations in which a massive interaction between dolphins (single or in groups) and trawls was documented. The group included at least four dolphins, as highlighted by the yellow circles in Fig. 1. However, the crew on board claimed that the whole group was larger (at least 6 or 7 dolphins).

**Fig. 2:**
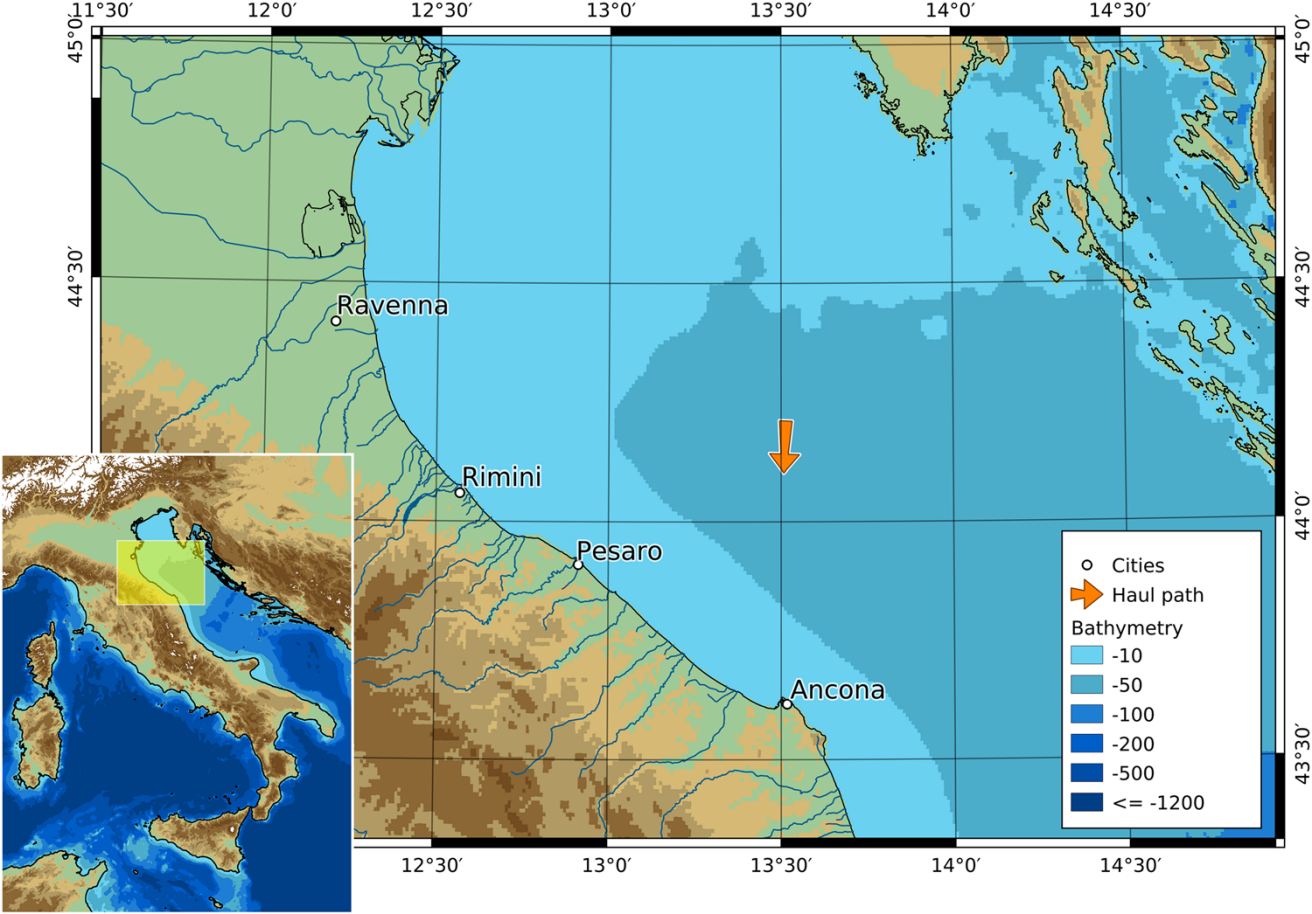
Map of the study area. The map highlights the location of the haul and, consequently, of the underwater acoustic recording. Maps were created using Qgis software (www.qgis.org).

The trawler (F/V Airone Bianco II, ITA000026441) fished on sandy bottoms ranging from 40 to 90 m in depth, a distance of 15 to 50 nautical miles off the coast of Ancona harbor, at an average fishing speed of approximately 3.2 knots. The towing started at 16:15 hours (CEST) at position 13.5138 E, 44.2111 N, depth 62.1 m, and ended at 18:47 hours at position 13.5079 E, 44.1026 N, depth 60.3 m (Fig. 2). The vessel used an Italian commercial bottom trawl (also known as “Americana”), made entirely of knotless polyamide netting. The dimensions of the trawl were approximately 60 m in length from wing tips to codend and a vertical opening during trawling of approximately 2 m, as described in^22^. The codend was made of the same net material with a 54 mm nominal mesh size. European hake *(Merluccius merluccius)*, shortfin squid *(Illex coindetii)*, European squid *(Loligo vulgaris)*, red mullet *(Mullus barbatus)*, and tub gurnard *(Chelidonichthys lucerna)* were the targeted fish species. A significant proportion of the fish in the catch escaped through the codend; this is probably the reason why the dolphins gathered around the codend^22^, as depicted in Fig. 1.

The UREC U384K autonomous underwater recorder (sampling rate = 192 kHz; resolution = 16 bits; bandwidth = 10 Hz–96 kHz; gain = 10x (20 dB)) developed by Dodotronic and Nauta and outfitted with a pre-amplified hydrophone, Sensor Technology SQ26-05, was utilized to collect data. Sensor Technology indicates that the hydrophone has a sensitivity of −193.5 dB re 1 V/μPa @ 20 °C between at least 1 Hz and 28 kHz. The sensitivity profile applicable to the entire SQ26 series is available at the link reported in^23^. However, additional studies also tested the sensitivity at higher frequencies. Palmero *et al*. report that the combination of this hydrophone SQ26-05 with the UREC U384K recorder have a sensitivity of −169 dB re 1 V/μPa up to 50 kHz^24^. The low-frequency response was not restricted during recording. The UREC U384K recorder was mounted directly on the net at the codend level, in the upper section, with a horizontal orientation towards the rear of the trawler. The hydrophone is at the end of the unit, and thus the long axis of the cylindrical hydrophone was pointing towards the codend. The omnidirectionality of the recorder/hydrophone system was only in the plane perpendicular to the UREC U384K housing and the hydrophone. The length and diameter of this cylinder-shaped unit were 35 cm and 10 cm, respectively. The average duration of a typical commercial haul is about 60 minutes; however, this haul was extended due to the persistent presence of dolphins. The recording session lasted a total of 120 minutes. The 120-minute recording was then processed to remove the initial 21 minutes and 40 seconds of noise-only recording. Thus, the actual signal, lasting 98 minutes and 20 seconds, was stored. This signal was further processed so that whistles and high-frequency pulsed vocalizations could be characterized. The following two approaches were employed.

### Whistle detection

**Fig. 3:**
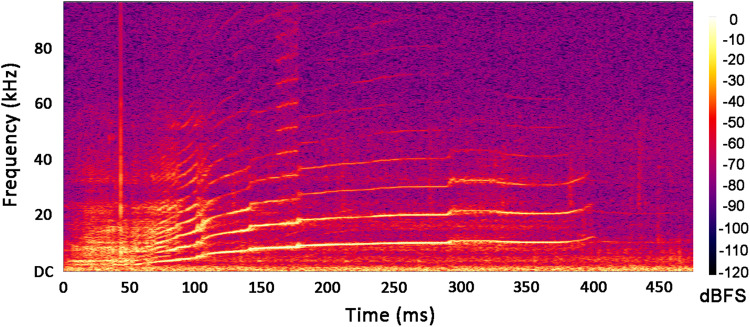
An example of the whistle acoustic signal’s frequency spectrum. This image corresponds to the whistle PNG file named 249_whistle_spectro.png stored in this dataset.

To visualize the frequency spectrum, the original signal was displayed in a 4-second time window using Audacity’s spectrogram interface (www.audacity.org) with a Hann window of 1,024 points. A trained and experienced passive acoustic monitoring (PAM) operator then visually inspected each audio spectrogram for the presence of whistles. Whistles were localized by visual inspection and manually centered in the 4-second window. Each individual whistle was then selected, cut to its effective length, saved as a WAV file, and reported in the dataset. A continuous contour on the 4-second spectrogram was identified as a single whistle. A fragmented contour consisting of several consecutive short elements was still acknowledged as a single whistle. The occurrence of multiple concurrent whistles from different dolphins in the same window was highlighted. Finally, the number of whistles in each window was counted, the location of the whistle was evaluated in terms of initial and final events, and the duration of the whistle was computed. An example of whistle identification is shown in Fig. 3. The yellow arrows indicate the start and end events as well as the duration of the whistles identified by the PAM operator.

### Analysis of high-frequency pulsed vocalizations

The recordings contain a large number of broadband, pulsed vocalizations. Two main categories of these sounds were identified: echolocation clicks and burst pulses. A computational process was devised to distinguish between the two categories in several different steps in accordance with the guidelines provided in Chapter 4 of the recent book “Passive Acoustic Monitoring of Cetaceans.”^25^

#### Noise filtering

The signal was high-pass filtered (Butterworth filter, eighth order, cut-off frequency: 10 kHz, roll-off: −48 dB) using the Audacity free audio editor (www.audacity.org) to remove the background noise from different sources such as the boat propeller, engine, etc.

#### Signal segmentation

The filtered signal, with a total duration of 98 minutes and 20 seconds, was split into ninety-eight 60-second segments and one 20-second segment using the SoX audio utility (https://sox.sourceforge.net/). Peak detection and classification were computed separately for each segment.

#### Signal-to-noise ratio (SNR) computation

The signal’s SNR was computed for each individual signal sample in accordance with Zimmer’s guidelines^25^. In order to reduce the computational load, the whole signal was split into a series of consecutive 60-second windows, and the procedure was computed in each window as follows:

1 $$SNR\left(t\right)=10\cdot log\frac{SIGNAL\left(t\right)}{NL}$$

where *SIGNAL(t)* is the value of the signal in each of the samples *t* of the 60-second window after the removal of the average value computed over all signal samples included in the 60-second window.

The noise level *(NL)* is expressed as the root mean square value computed in a single, specific 60-second segment of the recorder signal in which no dolphin vocalizations were detected:

2 $$NL=\sqrt{mean\left(NOISE{\left(t\right)}^{2}\right)}$$

where *NOISE(t)* is the signal value for each of the 60-second window samples *t* in which no dolphin vocalizations were detected. This segment was identified through visual inspection. NL is expressed as dBrms.

#### Peak detection

Initially, the average SNR value was computed along 2-ms windows along the signal. The presence of a peak was acknowledged only when the average SNR value exceeded a specific threshold value *Th* and both average SNR values in the previous and successive 2-ms windows were lower than *Th*. The value of *Th* was determined through a sensitivity analysis of the number of detected peaks to the SNR threshold value.

**Fig. 4:**
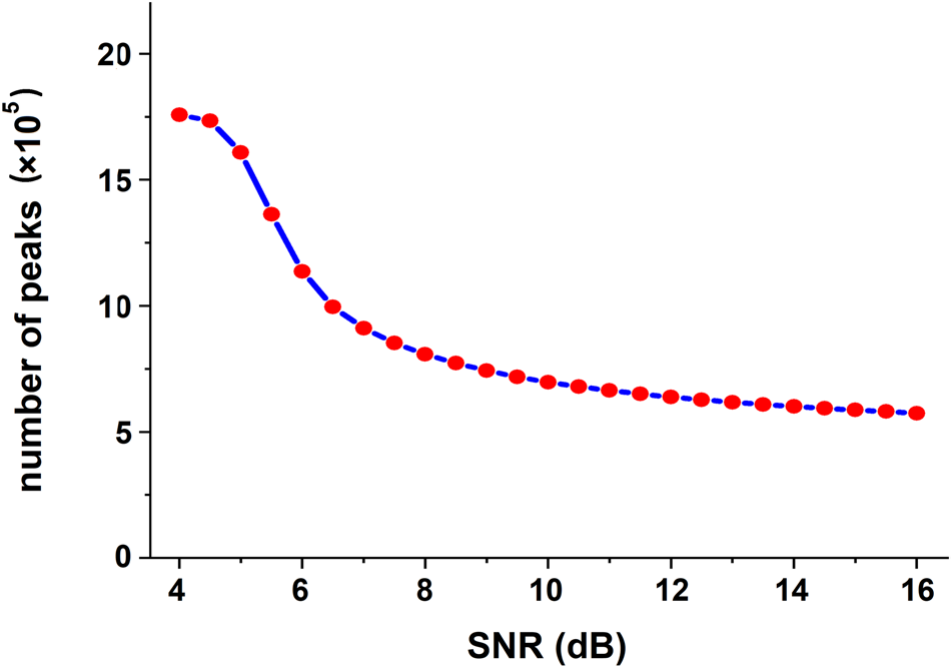
The number of detected peaks as a function of SNR threshold value. The red dots represent the total number of peaks detected using the current method for the different SNR values ranging from 4 dB to 16 dB with increments of 0.5 dB. The blue line is the interpolation curve.

**Fig. 5:**
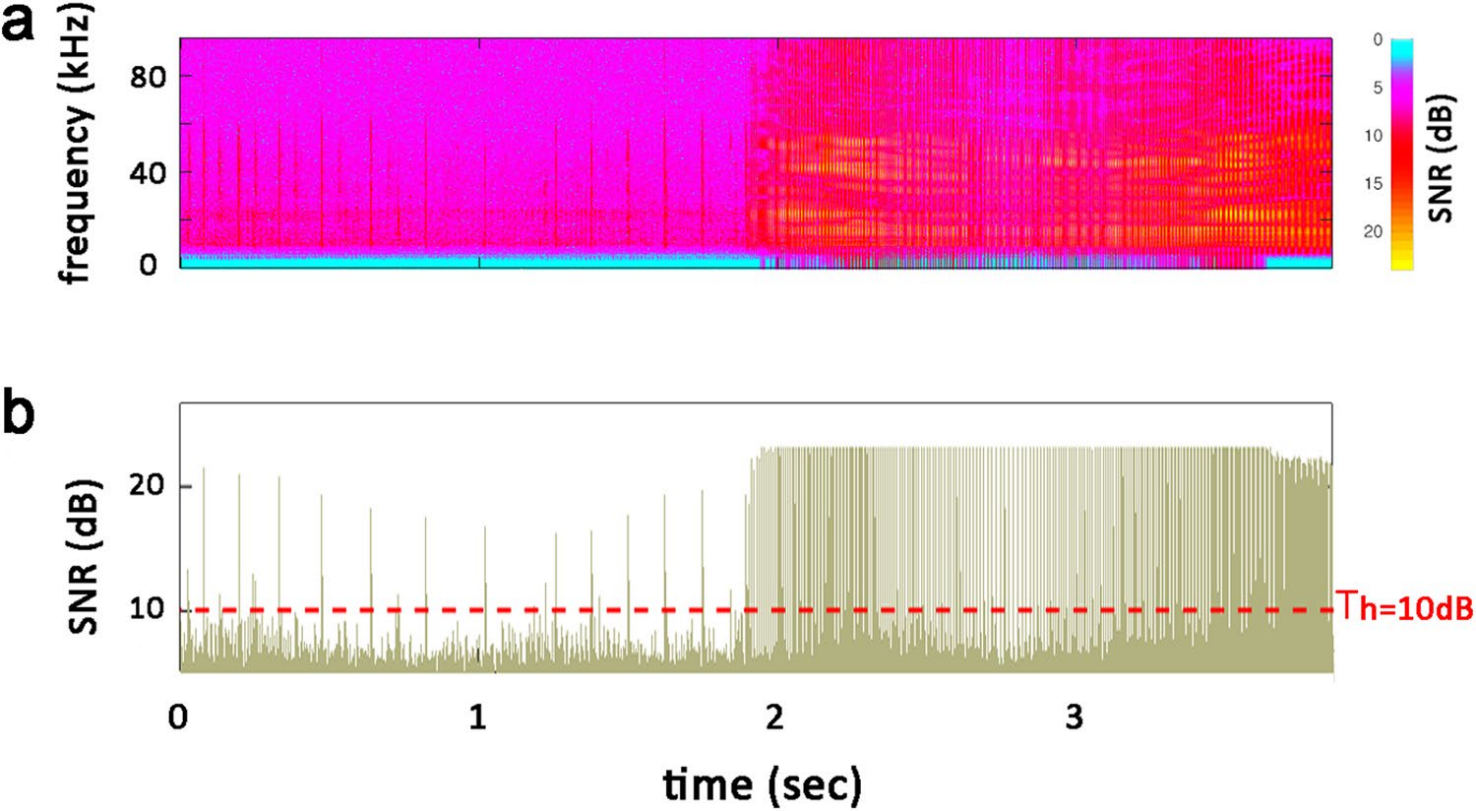
Peak detection mechanism. **(a**) Displays the signal’s spectrogram within a chosen window. Low signal levels are denoted by cyan, intermediate levels by magenta, high levels by red, and peak levels by yellow. (**b**) Illustrates peak detection by imposing the threshold *Th* on SNR values (red dashed line).

Specifically, SNR values from 4 dB to 16 dB (in increments of 0.5 dB) were tested. The results are reported in Fig. 4. The graph in Fig. 4 shows that for SNR = 4 dB, nearly 18 × 10^5^ peaks were detected in the entire recording. Starting from this value, the number of detected peaks decreases as a function of SNR following the decay curve highlighted in blue. The curve then seems to stabilize when SBR ≈ 10 dB. This indicates that after this value, SNR levels have minimal effect on the number of peaks. Consequently, this value was chosen as the threshold (*Th* = 10 dB). Finally, a script was written in GNU octave (version 6.4.0, Oct 30, 2021) to undertake this analysis in accordance with Chapter 4 of^25^. The basic algorithm code developed for peak detection is available at the figshare general-purpose open repository^26^. Figure 5 illustrates an example of peak detection.

#### Peak classification

A procedure was devised to classify the peaks identified in the previous section. The classification was based on the observation that click trains exhibit different values of the inter-click interval (*ICI*), which is defined as the absolute time distance between two consecutive peaks. In a depredation scenario in the vicinity of a trawl, dolphins will likely target the trawl and possibly escaping fish. In such a situation, dolphins would lock their sonar on these targets, adjust the *ICI* to the target’s distance, or generate feeding buzzes at close range. Thereafter, the *ICI* changes will be relatively smooth, transitioning from slow click trains (*IC I > *50–80 ms) in the exploring or search phase to 30 ms < *ICI < *50 ms in the first lock-on-target phase, then to 10 ms < *ICI < *40 ms during the approach phase, and finally drastically reducing the *ICI* in the feeding buzz to *ICI* < 10ms^18,27^.

**Fig. 6:**
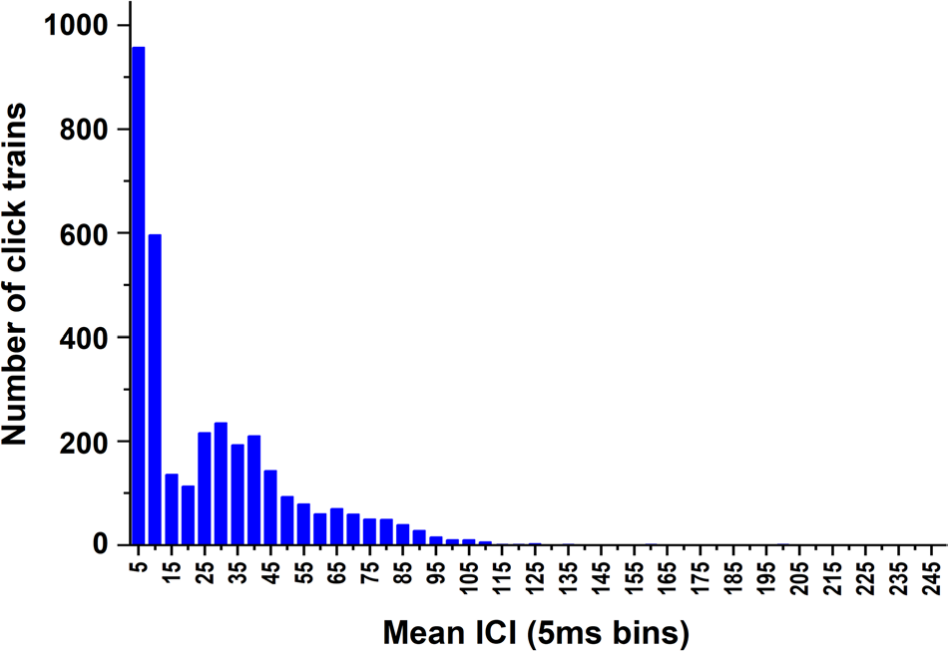
Peak classification. Histogram of click trains with mean inter-click intervals (*ICI*) in 5 ms bins.

Following the main points in Nuuttila’s study^18^, the current algorithm considered the output of the peak-detection routine and applied a filter based on peak amplitude and *ICI* to eliminate reverbs or overlapping click trains from more than one animal. Click trains were then identified based on an adaptive *ICI* threshold and the medium *ICI* value was computed in each click train. Results are provided in histograms, i.e., the number of clicks in *ICI* 5 ms bins (Fig. 6). A heavy dominance of mean *ICIs* in the 5 and 10 ms bins is highlighted, reflecting the dolphins’ close-range inspection of the net. A more spread-out and lower peak is detected in the 25–40 ms bins, reflecting exploring or approach click trains. Then, just few click trains with *ICI* above 110 ms are found.

## Data Records

This dataset consists of audio files in WAV format, comma-separated values (csv) files, text document files in txt format, and image files in Portable Network Graphic (PNG) format. The details of each file are provided below.

### WAV files

The recordings are provided as one complete file and a folder containing 303 audio segments:

- **full_recording.wav** is a single raw audio WAV file (sampling rate = 192 kHz; resolution = 16 bits, encoding Microsoft PCM) containing the complete, unedited recording of the bottlenose dolphins’ emissions. The duration of the entire file is 98 minutes and 20 seconds;
- **whistle_segments** is a folder containing 303 audio WAV files extracted from the full_recording.wav file. Each of the 303 files contains a single whistle or a group of multiple whistles (when multiple dolphins were concomitantly emitting) and is stored as an individual WAV file with a sequential name (001_whistle.wav, 002_whistle.wav, 003_whistle.wav, etc.).

### CSV files

One comma-separated values (CSV) file is included in the dataset to report audio-emission detection and classification summary results.

**results.csv** is a csv file containing quantitative data regarding the emission-peak detection and classification of each of the ninety-eight 60-second and one 20-second segments used to identify the high-frequency pulsed vocalizations. Information regarding the presence of single or multiple whistles is also reported. The file is structured with the following fields:

1. *Block#* represents the sequential number of each one-minute segment;
2. *Peak number* reports the total number of peaks identified by the “Peak detection” procedure in each segment;
3. *Train number* reports the total number of click trains identified in each segment;
4. *ICImin* reports the minimum value of the *ICI* in the segment under consideration;
5. *ICImax* reports the maximum value of the *ICI* in the segment under consideration;
6. *ICImean* reports the mean value of the ICI in the segment under consideration;
7. *Whistle* reports the number of single and multiple whistles detected in the segment under consideration;
8. *Multiple W* indicates whether the block under consideration contains multiple whistles (YES) or otherwise (NO).

### TXT files

Two txt files were included in the database to facilitate the interpretation of the audio signals by means of the Audacity software (direct import of.txt files).

**whistles.txt** is a tab-separated text file containing the following data for each detected whistle:

1. whistle start time expressed in seconds as the time stamp in the original full recording, counting from the start of the recording;
2. whistle end time expressed in seconds as the time stamp in the original full recording, counting from the start of the recording;
3. classification tag: W for single whistle and MW for multiple whistles;
4. whistle signal quality classes (1, 2, and 3) (see “Technical Validation” section).

**clicks.txt** is a tab-separated text file containing the following timing data for each detected high-frequency pulsed vocalization. The minimum time interval between two click trains is 250 ms:

1. click train start time expressed in seconds as the relative time stamp in the original full recording;
2. click train end time expressed in seconds as the relative time stamp in the original full recording;
3. mean *ICI* 5 ms bins corresponding to each click train.

All the parameters reported in the whistles.txt and clicks.txt files are also depicted as labels that can be imported directly into the Audacity software for visualization (file → import → labels).

### PNG files

The **whistle_spectrograms** folder contains 303 PNG files. The PNG files depict the spectrograms of the 303 whistles contained in the whistle_segments folder and are stored as individual PNG files with a sequential name corresponding to the whistle name (001_whistle_spectro.png, 002_whistle_spectro.png, 003_whistle_spectro.png, etc.).

The dataset is available via an unrestricted repository at figshare^28^.

### Data limitations

These data were recorded with a sampling rate of 192 kHz. It has been reported that peak frequencies of bottlenose dolphin vocalizations may reach up to 150 kHz^14,20,29^. A higher sampling rate will extend the frequency range, allowing for greater discrimination between different types of vocalizations^30^. However, a higher sampling rate would require a more broadband hydrophone than the one used in this study. Consequently, one could argue that the present sampling rate of 192 kHz is insufficient to describe the full bandwidth of vocalization signals. However, the optimal sampling frequency for recording bottlenose dolphin vocalizations remains a matter of debate. In general, it is important to consider the limitations and challenges associated with high sampling rates, such as higher equipment costs and larger data storage requirements. In the intended recording context, higher costs would only be related to the purchase of a higher-performing hydrophone, as the recorder and memory are already suitable in the current setup. Moreover, it is acknowledged that sampling frequencies can vary depending on the type of vocalization being recorded. Different studies have reported that the frequency content of signature whistles ranges from 1 to 30 kHz^31^. Therefore, a sampling frequency of 192 kHz is totally suitable for identifying and classifying dolphin whistles based on their spectral characteristics.

Although it has been reported that the on-axis echolocation signals occur with bimodal peak frequencies in the 60–90 and 110–140 kHz ranges^29^, a dominant frequency ranging from 40 kHz to 80 kHz was identified in a large collection of underwater sounds (clicks) from six specimens of *Tursiops truncatus*^32,33^. An interesting study analyzed the median peak and center frequencies of bottlenose dolphin echolocation clicks to determine their spectral characteristics^34^. Three different recording instruments were utilized with three different sampling rates (192 kHz, 200 kHz, and 480 kHz). The results revealed that the median peak frequency ranged from 27.2 to 35.6 kHz, and the median center frequency ranged from 34.0 to 39.8 kHz. Moreover, acquisition with a sampling rate of 192 kHz revealed a peak frequency distribution comparable to that of 480 kHz recordings. The higher sampling rate exhibited no significant peaks at higher frequencies. Notably, high-frequency echolocation signals occur primarily when vocalizations are recorded along the axis of the dolphin’s transmitting beam^15^. Outside of the beam, the low-frequency components experience less attenuation and appear in the frequency spectrum^35^. Therefore, low-frequency components are expected to be predominant when free-ranging dolphin vocalizations are recorded since they are mainly off-axis of the transmitting beam^36^. In the current study, bottlenose dolphin vocalizations were recorded with no regard to specific distance or orientation from the hydrophone. Based on these considerations, it was determined that the frequency of 48 kHz could be effective for recording the entire acoustic repertoire of *Tursiops truncatus*, including echolocation signals, in studies analyzing the occurrence or distribution of individuals in a population or at a specific location when the presence or absence data are sufficient^36^. These observations indicate that the present database recorded at 192 kHz could be suitable for the majority of dolphin vocalization analyses. To support this, it is important to note that numerous studies on the high-frequency echolocation clicks of bottlenose dolphins utilized this (or a lower) sampling frequency^37–39^. However, for other purposes, such as the fine identification or classification of dolphin species, the recording of the full bandwidth is recommended.

Moreover, the sensitivity curve of the SQ26-05 hydrophone (see “Data collection” section) indicated a reduction in sensitivity between 30 kHz and 40 kHz (≈−5 dB). This presumably affects the detection of the reported 35–40 kHz median center frequency^34^. The sensitivity then returns to the previous value of up to 50 kHz. No sensitivity data are reported for higher frequencies. Thus, the hydrophone’s sensitivity in this frequency range may be affected by a reduced sensitivity. Users are advised to take this issue into consideration when utilizing the current dataset.

**Fig. 7:**
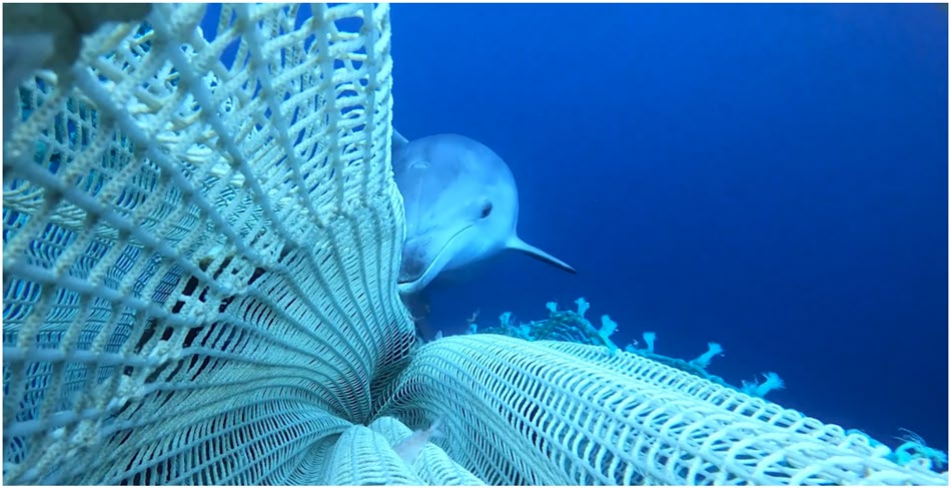
An example of dolphin interaction with the trawl net.

The present recordings were collected during fishing operations as part of the experimental campaign conducted within the framework of the EU Life Delfi project, which seeks to introduce new technical solutions to control and reduce interactions between marine mammals and commercial fishing activities. Specifically, one of the main goals is to assemble an acoustic device capable of continuously recording underwater sounds, identifying the presence of dolphins, and then driving a pinger device to discourage dolphins from approaching the trawl net. The purpose of these recording sessions was to acquire dolphin vocalization signals for the pinger project. Therefore, the hydrophone was positioned directly on the trawl net to optimize the recording, especially the long-distance recordings that are more useful for the advance detection of dolphin presence. Figure 7 illustrates that during the recording of the depredation event, one or more dolphins approached the hydrophone very closely. In these instances, the sound pressure level produced by the dolphin’s vocalizations exceeds the hydrophone’s dynamic range, causing the signal to saturate. Moreover, dolphins could come so close as to bite or bump into the hydrophone. The resultant impact may temporarily interrupt the recording or generate a high-amplitude signal that saturates the hydrophone. This phenomenon, which has also been identified in the literature, could result in signal overload, waveform distortion, and no-sound sections in some parts of the proposed dataset^40^. This was the first attempt to record dolphins in this characteristic context, and some gain settings may have been suboptimal. This also contributes to the signal overload. Social burst pulse signals (creaks, squawks, and short burst pulses) often have significantly more energy <20 kHz than echolocation clicks^41^, distinguishing them from echolocation low-*ICI* click trains. The overloading may hinder the ability to identify these vocalizations. Overloaded parts of the signal were included in the current dataset to enable the analysis of possible vocalizations that were not affected by this phenomenon.

This classification of clicks was performed on a filtered signal (high-pass filter, cut-off frequency = 10 kHz). It has been reported that social burst pulse sounds often contain considerable energy at low frequencies (<3 kHz)^42^. It is not possible to detect such vocalizations with the current setup due to the essential filtering process employed to eliminate background noise. This should be regarded as a limitation of the click classification procedure. As the recording stored in the repository is not filtered, users may employ advanced filtering techniques to extract the low-frequency social burst pulse sounds.

As previously discussed, the current dataset may be beneficial for advancing our understanding of dolphin vocalizations during depredation events. In addition to the traditional analysis of dolphin sounds conducted in this study, the dataset could be used to undertake a PAM analysis (as provided by the classic PAM system, Chelonia’s C-PODs, and F-PODs) to calculate abundance, distribution, and seasonal and diel variations^42^. Moreover, it could be useful as a proposed format for dealing with similar datasets. To emphasize the applicability of the data, comparable control data from the same area with a group of dolphins of a comparable size could be relevant. Similarly, more data from other events of this type (such as other depredation or interaction types^43^, such as with gillnets and pelagic trawls) would enhance the dataset’s generalizability. Although additional experimental campaigns have been scheduled for this purpose, such data are not currently available. This may be viewed as an additional limitation of the study. Moreover, only one hydrophone position was used in the current setup, and alternative positions that may be suitable for triggering the future interactive pinger were not investigated. This potential weakness will be addressed in future studies.

Following a comprehensive discussion of the proposed dataset’s limitations, primarily attributable to the fact that this was our first attempt to record dolphins during depredation, the following are the main useful recommendations concerning essential technical modifications and enhancements for future recordings of the depredation event: (1) employing a hydrophone with linear sensitivity (no loss) across the entire frequency range of dolphin vocalizations (0–150 kHz);^14,20,29^ (2) increasing the sampling frequency to capture the full bandwidth of dolphin vocalizations which allows to correctly record high-frequency echolocations clicks and thus to help revealing scanning behavior, and facilitating discrimination between overlapping click trains^44^. It would also allow for selective triggering, based on *ICI* and/or frequency content in the clicks; (3) conducting a number of pilot recordings to determine the optimal settings (including gain) for obtaining good quality recordings and avoiding overloads; and (4) applying a high-pass filter with a cut-off frequency of 1–2 kHz while recording to remove most of the noise from the propeller and engine.

## Technical Validation

To verify the reliability of the dataset, the signal-to-noise ratio (SNR) as calculated in Eq. 1 was used to analyze the quality of the reported whistles. First, a trained and experienced PAM operator visually inspected all 303 whistle spectrograms before dividing them into three classes according to the classification introduced in^13^ and also adopted in^8^. Class 3 includes segments in which the whistle is prominent; Class 2 includes segments in which whistle detection is clear and unambiguous; and Class 1 includes whistles of lower quality (weak signals). According to the literature^13^, classes 2 and 3 whistles were recognized as being of high quality. The mean SNR value and duration (±standard error, SE) were then computed for the whistle spectrograms in each class. The results are reported in Table 1.

**Table 1:** Characteristics of classification classes based on whistle quality.

Whistle quality class	Number of whistles	Mean duration ± SE (ms)	MeanSNR ± SE (dB)
1	122	484 ± 31	7.09 ± 0.11*
2	104	776 ± 81	8.00 ± 0.21^§^
3	77	759 ± 110	9.29 ± 0.31

SNR means signal-to-noise ratio. SE is standard error. *means statistically different from classes 2 and 3 (*p* < 0.05). ^§^means statistically different from class 3 (*p* < 0.05).

As expected, the SNR in the three whistle classes increases as the quality class (from 1 to 3) increases. This result further supports the reliability of the present dataset. Indications of whistle quality class are provided in the whistles.txt file for each whistle.

Despite the above-mentioned limitations about the recording of high-frequency vocalizations, the distribution of average *ICIs* of click trains is in agreement with what reported by Nuuttila’s *et al*.^18^. As shown in Fig. 6, it is characterized by three peaks: a first distinct peak of very short *ICIs* (<5 ms), a further higher peak, and a third less pronounced peak comparable to the first one. Then, a slow decay is detected for higher *ICIs* up to 500 ms. It is worth highlighting that most of click trains is characterized by average *ICI* shorter than 200 ms and only a minimum part presents a higher average *ICI*.

Although the standard procedure would have been to make one or more pilot recordings to determine the optimal settings for good quality recordings, this recording is the first and only one made. Only one recording was made due to the exceptional nature of the event, which involved the presence of several dolphins near the fishing net. This resulted in the overload of some segments of the recording and certain prioritized vocalizations (i.e., the click trains) due to settings that were not always suitable for the experimental condition. This factor should be taken into account during the analysis. Therefore, to ensure the recording’s usefulness, an analysis was conducted to identify the parts where the signal was overloaded. The analysis was performed using two different thresholds to identify overloads: 99% and 90% of the signal peak. The results obtained with the 99% threshold indicate that only 6356601 samples (0.56% of the total samples) exceeded the threshold and were subsequently overloaded. The results obtained with the more selective 90% threshold indicate that 15689091 samples (1.38% of the total samples) were overloaded. Therefore, only a small percentage of the signals are affected by overload. This result is probably attributable to the fact that only the high-amplitude, short clicks are clipped. The whistles have a much lower amplitude and were never clipped. Therefore, they can be analyzed even when they occur within clipped click trains.

## Data Availability

The basic algorithm code developed for peak detection is available at the general-purpose open repository figshare^26^. The aim of the algorithm is to achieve a tool able to provide an overview of the richness of the dataset content and its potentiality. Otherwise, to provide a robust and detailed quantitative analysis of the different vocalizations included in the dataset is beyond the purposes of the present data descriptor.

## References

[1] Jog K, Sutaria D, Diedrich A, Grech A, Marsh H (2022). Marine mammal interactions with fisheries: review of research and management trends across commercial and small-scale fisheries. Front. Mar. Sci..

[2] Bonizzoni S, Hamilton S, Reeves RR, Genov T, Bearzi G (2022). Odontocete cetaceans foraging behind trawlers, worldwide. Rev Fish Biol Fisheries.

[3] Tixier P (2020). When large marine predators feed on fisheries catches: global patterns of the depredation conflict and directions for coexistence. Fish Fish. (Oxf).

[4] Maccarrone V (2014). Economic assessment of dolphin depredation damages and pinger use in artisanal fisheries in the archipelago of Egadi islands (Sicily). Turkish J. Fish. Aquat. Sci..

[5] Northridge, S. P. An updated world review of interactions between marine mammals and fisheries. *FAO Fish. Tech. Pap*. **251** (1991).

[6] Díaz López B (2011). Whistle characteristics in free-ranging bottlenose dolphins (Tursiops truncatus) in the Mediterranean Sea: Influence of behaviour. Mamm. Biol..

[7] Luís AR (2021). Vocal universals and geographic variations in the acoustic repertoire of the common bottlenose dolphin. Sci. Rep..

[8] Marley S, Erbe C, Kent C (2017). Underwater recordings of the whistles of bottlenose dolphins in Fremantle Inner Harbour, Western Australia. Sci. Data.

[9] Gregorietti M (2021). Acoustic Presence of Dolphins through Whistles Detection in Mediterranean Shallow Waters. J. Mar. Sci. Eng..

[10] Caldwell, M. C., Caldwell, D. K. & Tyack, P. L. Review of the signature-whistle-hypothesis for the Atlantic bottlenose dolphin, Tursiops truncatus. *The Bottlenose Dolphin*. (San Diego: Academic Press, 1990).

[11] Lammers, M. O. & Oswald, J. N. Analyzing the acoustic communication of dolphins. *Dolphin Communication and Cognition Past, present and Future*. (Cambridge, MA: MIT Press, 2015).

[12] Janik V, Slater P (2000). The different roles of social learning in vocal communication. Anim. Behav..

[13] Heiler J, Elwen SH, Kriesell HJ, Gridley T (2016). Changes in bottlenose dolphin whistle parameters related to vessel presence, surface behaviour and group composition. Anim. Behav..

[14] Hernandez EN, Solangi M, Kuczaj SA (2010). Time and frequency parameters of bottlenose dolphin whistles as predictors of surface behavior in the Mississippi Sound. J. Acoust. Soc. Am..

[15] Au W. W. L. *The Sonar of Dolphins*. (New York, NY: Springer New York, 1993).

[16] Au WWL (2004). Echolocation signals of wild dolphins. Acoust. Phys..

[17] Díaz López, B. & Shirai, J. A. B. Mediterranean common bottlenose dolphin’s repertoire and communication use. *Dolphins: Anatomy, Behavior and Threats*. (New York, NY: Nova Science Publishers; 2009).

[18] Nuuttila HK (2013). Identifying Foraging Behaviour of Wild Bottlenose Dolphins (Tursiops truncatus) and Harbour Porpoises (Phocoena phocoena) with Static Acoustic Dataloggers. Aquatic Mammals.

[19] Lammers MO, Au WWL, Herzing DL (2003). The broadband social acoustic signaling behavior of spinner and spotted dolphins. J. Acoust. Soc. Am..

[20] Jones B, Zapetis M, Samuelson MM, Ridgway S (2020). Sounds produced by bottlenose dolphins (Tursiops): A review of the defining characteristics and acoustic criteria of the dolphin vocal repertoire. Bioacoustics.

[21] Mellinger DK, Stafford KM, Moore SE, Dziak RP, Matsumoto H (2007). An overview of fixed passive acoustic observation methods for cetaceans. Oceanography.

[22] Petetta A (2020). Estimating selectivity of experimental diamond (T0) and turned mesh (T90) codends in multi-species Mediterranean bottom trawl. Mediterr. Mar. Sci..

[23] SQ26-05 hydrophone, Sensor Technology, Nauta Scientific website Accessed June 05, 2023 https://www.nauta-rcs.it/EN/Hydrophones/SensorTech/ (2023).

[24] Palmero, S. et al. Towards automatic detection and classification of orca (Orcinus orca) calls using cross-correlation methods. *Mar. Mammal Sci*. 1–18 (2022)

[25] Zimmer, W. M. X. *Passive Acoustic Monitoring of Cetaceans* (Cambridge University Press, Cambridge, 2011).

[26] De Marco R (2023). figshare. Software..

[27] Verfuß UK, Miller LA, Pilz PK, Schnitzler HU (2009). Echolocation by two foraging harbour porpoises (Phocoena phocoena). J Exp Biol..

[28] Di Nardo F (2022). figshare.

[29] Akamatsu T, Wang D, Nakamura K, Wang K (1998). Echolocation range of captive and free-ranging baiji (Lipotes vexillifer), finless porpoise (Neophocaena phocaenoides), and bottlenose dolphin (Tursiops truncates). J. Acoust. Soc. Am..

[30] Wisniewska DM, Johnson M, Madsen PT, Teilmann J (2016). High rates of vessel noise disrupt foraging in wild harbour porpoises (Phocoena phocoena). Proceedings of the Royal Society B: Biological Sciences.

[31] Janik VM, Sayigh LS (2013). Communication in bottlenose dolphins: 50 years of signature whistle research. J Comp Physiol A Neuroethol Sens Neural Behav Physiol..

[32] Kamminga C, Beitsma GR (1990). Investigations on cetacean sonar 9. Remarks on dominant sonar frequencies from Tursiops truncates. Aquat. Mamm..

[33] Kamminga C, Cohen Stuart A, Silber GK (1996). Investigations on cetacean sonar XI: Intrinsic comparison of the wave shapes of some members of the Phocoenidae family. Aquat. Mamm..

[34] Baumann-Pickering S, Wiggins SM, Hildebrand JA, Roch MA, Schnitzler HU (2010). Discriminating features of echolocation clicks of melon-headed whales (Peponocephala electra), bottlenose dolphins (Tursiops truncatus), and Gray’s spinner dolphins (Stenella longirostris longirostris). J Acoust Soc Am..

[35] Finneran JJ (2014). High resolution measurement of a bottlenose dolphin’s (Tursiops truncatus) biosonar transmission beam pattern in the horizontal plane. J. Acoust. Soc. Am..

[36] Romeu B (2021). Low-frequency sampling rates are effective to record bottlenose dolphins. R Soc Open Sci..

[37] Luís AR, Couchinho MN, dos Santos MEA (2016). Quantitative Analysis of Pulsed Signals Emitted by Wild Bottlenose Dolphins. PLoS ONE.

[38] Roch MA (2011). Classification of echolocation clicks from odontocetes in the Southern California Bight. J Acoust Soc Am..

[39] Tellechea J (2020). Echolocation inter-click interval variation among specific behaviours in free-ranging bottlenose dolphins from the coast of Uruguay. JCRM.

[40] Au, W. W. L. & Hastings, M. C. *Principles of Marine Bioacoustics* (New York: Springer, 2008).

[41] Blomqvist, C. & Amundin, M. *High-Frequency Burst-Pulse Sounds in Agonistic/Aggressive Interactions in Bottlenose Dolphins, Tursiops truncatus*. In: Thomas, J. A., Moss, C.F., Vater, M. editors. Echolocation in Bats and Dolphins (Chicago, USA.: The University of Chicago Press. p 425–431 2004).

[42] Amundin M (2022). Estimating the abundance of the critically endangered Baltic Proper harbour porpoise (Phocoena phocoena) population using passive acoustic monitoring. Ecol. Evol..

[43] Königson S (2022). Will harbor porpoises (Phocoena phocoena) be deterred by a pinger that cannot be used as a “dinner bell” by seals?. Mar. Mammal Sci..

[44] Starkhammar J (2011). Separating overlapping click trains originating from multiple individuals in echolocation recordings. J. Acoust. Soc. Am..

